# Polymyxin B-immobilized fiber column hemoperfusion mainly helps to constrict peripheral blood vessels in treatment for septic shock

**DOI:** 10.1186/s40560-015-0080-9

**Published:** 2015-03-20

**Authors:** Makiko Sugiura, Chieko Mitaka, Go Haraguchi, Makoto Tomita, Naohiko Inase

**Affiliations:** Department of Respiratory Medicine, Tokyo Medical and Dental University, Tokyo, Japan; Department of Critical Care Medicine, Tokyo Medical and Dental University Graduate School, Tokyo, Japan; Division of Intensive Care Unit, New Tokyo Hospital, Chiba, Japan; Medical Clinical Research Center, Tokyo Medical and Dental University, Tokyo, Japan

**Keywords:** Severe sepsis, Septic shock, Polymyxin B, Endotoxin, Hemodynamics, Hemoperfusion, Inotropic score

## Abstract

**Background:**

Polymyxin B-immobilized fiber column hemoperfusion (PMX) has been reported to be effective for patients with septic shock. It remains unclear, however, how the efficacy of PMX varies according to the characteristics and underlying conditions of the patients treated. The objective of the present study was to clarify the factors that result in clinical efficacy of PMX treatment.

**Methods:**

We retrospectively investigated 78 consecutive patients with severe sepsis or septic shock who underwent PMX treatment. We reviewed the demographic data, routine biochemistry, microbiological data, infection focus, Acute Physiology and Chronic Health Evaluation (APACHE) II score, Sequential Organ Failure Assessment (SOFA) score, change in mean arterial pressure (MAP), inotropic score, vasopressor dependency index, plasma levels of endotoxin and lactate, PaO_2_/F_I_O_2_ ratio, and survival time. We also divided the patients into two groups for comparison, namely, those whose inotropic scores improved after PMX treatment (improvement group) and those whose inotropic scores did not improve (non-improvement group).

**Results:**

The inotropic score and the vasopressor dependency index significantly decreased from 18.1 to 9.9 (*p* < 0.05) and from 0.27 to 0.14 (*p* < 0.05), respectively, after PMX treatment in the overall study population, while no significant change in the PaO_2_/F_I_O_2_ ratio was observed (*p* = 0.96). The inotropic score at pre-PMX treatment was significantly higher in the improvement group than in the non-improvement group (*p* < 0.01). The improvement of the PaO_2_/F_I_O_2_ ratio after PMX treatment was significant in the improvement group (*p* < 0.05).

**Conclusions:**

The improvement group’s inotropic score was higher, because of peripheral blood vessels dilatation and requirement for more catecholamines. Therefore, our study suggests that PMX treatment is particularly useful for improving hemodynamics in septic shock patients with excessively dilated peripheral blood vessels.

## Background

Septic shock is still a serious event associated with high mortality and multiple organ failure in spite of advances in intensive therapy techniques and the introduction of new drugs [1]. When a patient goes into sepsis, endotoxin (lipopolysaccharide; LPS), a component of the outer membrane of Gram-negative bacteria, induces a very strong acute inflammatory response, stimulates immunocompetent cells such as macrophages, and activates inflammatory cytokines, platelet-activating factor, and nitric oxide synthase. These changes often go on to cause fatal circulatory failure and multiple organ failure. Polymyxin B-immobilized fiber column hemoperfusion (PMX) is a method to adsorb the circulating endotoxin in blood by direct extracorporeal hemoperfusion without exerting toxicity [2]. This fiber column covalently immobilizes polymyxin B, a cyclic polypeptide antibiotic, and binds to endotoxin [3]. The column contains a sheet of braided fibers composed of polystyrene on the outer portion of the sheet and polypropylene on the inner portion. Polystyrene fibers covalently bind to polymyxin B, a strong LPS adsorber. Lipid A of endotoxin binds to the amino group of polymyxin B via ionic bonding, or the hydrophobic moiety of LPS binds to the hydrophobic chain of polymyxin B via hydrophobic bonding [3]. Hence, the endotoxin can be effectively removed. Recent studies have also demonstrated the ability of PMX to remove endogenous cannabinoids [4]. Other papers, meanwhile, report that the endotoxin removal by PMX helps to reduce several other mediators such as interleukin (IL)-6 [5,6], tumor necrosis factor (TNF)-α [7], and tissue inhibitor of metalloproteinase 1 [8,9]. PMX was first developed in 1994 and has been widely used in patients with septic shock in Japan. The method is reportedly effective for both increasing the mean arterial pressure (MAP) [10,11] and improving PaO_2_/F_I_O_2_ in patients with septic shock [10]. Yet, in spite of these reported benefits from PMX treatment, the treatment has not conferred sufficient effects in patients with severe sepsis. Hence, it remains unclear how the efficacy of PMX treatment varies according to the characteristics and underlying conditions of the patients treated. The objective of the present study was to clarify the factors that result in clinical efficacy of PMX treatment.

## Methods

This was a retrospective observational study conducted in the intensive care unit (ICU) of Tokyo Medical and Dental University between April 2004 and July 2013. The study was approved by our Institutional Ethics Committee after the patients or their families gave informed consent to a review of patient records. We retrospectively reviewed demographic data, routine biochemistry, microbiological data, infection focus, the Acute Physiology and Chronic Health Evaluation (APACHE) II score, the Sequential Organ Failure Assessment (SOFA), and the survival time of 78 consecutive patients with severe sepsis or septic shock. The inclusion criteria for PMX treatment were severe sepsis or septic shock with an increased plasma endotoxin concentration or suspected Gram-negative infection. Severe sepsis was defined as sepsis plus sepsis-induced acute organ dysfunction or tissue hypoperfusion secondary to documented or suspected infection. Septic shock was defined as severe sepsis plus hypotension not reversed with adequate fluid resuscitation [12].

The PMX treatment was administered by the following method. A Toraymyxin 20-R cartridge (Toray Industries, Tokyo, Japan) was washed by perfusion with 4 L of 0.9% saline. After inserting a double-lumen catheter into a central vein according to the Seldinger method, blood was drawn from the proximal port, perfused through the Toraymyxin 20-R cartridge, and returned to the vein through the distal port of the catheter. The perfusion was performed at a rate of 80 to 100 mL/min using nafamostat mesilate protease inhibitor (Torii Pharmaceuticals, Co., Ltd., Tokyo, Japan) as an anticoagulant. The PMX treatment was commenced in the ICU within 24 h after diagnosis of severe sepsis or septic shock and extended for as long as possible beyond 2 h. The duration of PMX treatment was determined by consensus at a conference of attending physicians, intensivists, and nephrologists. This duration varied according to both the time of commencement of treatment and the arrangements of the staff in the Department of Blood Purification [13].

The assay for endotoxin was performed with separated plasma from heparinized whole blood samples centrifuged at 3,000 rpm for 40 s. The high-sensitivity assay was performed by a kinetic turbidimetric Limulus assay using a MT-358 Toxinometer (Wako Pure Chemical Industries, Ltd., Osaka, Japan), a device theoretically capable of measuring with an accuracy of up to 0.01 pg/mL. This Limulus assay tests for a specific endotoxin and is confirmed not to cross-react with β-glucan [14]. The cutoff endotoxin level for the diagnosis of sepsis is 1.1 pg/mL. Changes in MAP and the PaO_2_/F_I_O_2_ ratio were also evaluated. The inotropic score was calculated as (dopamine dose × 1) + (dobutamine dose × 1) + (adrenaline dose × 100) + (noradrenaline dose × 100), where all doses are expressed as microgram per kilogram per minute [10]. We also calculated the vasopressor dependency index, which was the ratio of inotropic score to MAP, because a dose-response relationship between vasopressor dose and MAP has been used as the degree of hemodynamic impairment [10]. All clinical data were recorded at baseline, at completion of the PMX treatment, and at the time of death or hospital discharge.

The hemodynamics and respiratory status were assessed before and immediately after the PMX treatment. Subsequently, all patients were separated into two groups for analysis: improvement and non-improvement groups. When the inotropic score before the PMX treatment minus that after the PMX treatment was greater than 0, patient was allocated to improvement group. When the inotropic score before the PMX treatment minus that after the PMX treatment was less than or equal to 0, patient was allocated to non-improvement group. The reason why we select the factor inotropic score is that this factor significantly contributes to improve outcome in 28 days survival group by preliminary examination.

### Statistical analysis

Continuous variables are presented as means and 95% confidence intervals (CIs). Comparisons of continuous variables between the two groups were conducted with the Mann-Whitney *U* test. Inter-group comparisons of categorical variables such as sex, history of diabetes, or history of corticosteroid therapy were conducted with the Fisher’s exact test. Comparisons among different points (baseline and the time of finished the PMX treatment) within groups were performed using a Wilcoxon signed rank sum test. The duration of survival was calculated from the date of ICU admission to the date of death or the date of hospital discharge and analyzed by a log-rank test. All statistical analyses were performed with EZR (Saitama Medical Center, Jichi Medical University, Saitama, Japan), a graphical user interface for R (The R Foundation for Statistical Computing, Vienna, Austria). More precisely, EZR is a modified version of R commander designed to add statistical functions frequently used in biostatistics. A *p* value of less than 0.05 was considered statistically significant.

## Results

The characteristics of all patients are summarized in Table 1. The median age was 70 years, and 50 of the 78 patients were male. Median total PMX treatment time was 1,456 min. The sources of infection included abdominal infection, bloodstream infection, respiratory infection, urinary tract infection, and others (unknown origin). Suspected causative microorganisms were shown in Table 1. Gram-negative rods (GNR) include *Escherichia coli*, *Klebsiella pneumoniae*, *Psudomonas aeruginosa*, *Serratia marcescence*, *Acinetobacter baumanni*, *Stenotrophomonas maltophilia*, *Enterobacter cloacae*, *Achromobacter xylosoxidans*, and *Bacteroides fragilis*; Gram-positive cocci (GPC) include MRSA (Methicillin-resistant *Staphylococcus aureus*), *Enterococcus faecalis*, *Enterococcus faecium*, and group *A streptococcus*; and Gram-positive rods (GPR) include *Corynebacterium*. All patients were followed-up until death (*n* = 46) or hospital discharge (*n* = 32). Table 2 shows the changes in the inotropic score, the vasopressor dependency index, and the PaO_2_/F_I_O_2_ ratio from baseline to post-PMX in all patients. The inotropic score and the vasopressor dependency index significantly decreased from 18.1 to 9.9 (*p* < 0.05) and from 0.27 to 0.14 (*p* < 0.05), respectively, after PMX treatment, while the PaO_2_/F_I_O_2_ ratio showed no significant change (*p* = 0.96). Table 3 shows the characteristics of the improvement and non-improvement groups in this study. There were no significant differences between the two groups in organ dysfunction, MAP before the PMX treatment, chronic illness, the endotoxin level before and after the PMX treatment, the times of PMX therapy, total PMX treatment time, or the PaO_2_/F_I_O_2_ ratio before the PMX treatment. The inotropic score and the vasopressor dependency index before the PMX treatment, however, were significantly higher in the improvement group than in the non-improvement group. This meant that the improvement group required large doses of catecholamine before the PMX treatment. The PaO_2_/F_I_O_2_ ratio was also significantly improved after PMX treatment in the improvement group. The median survival time was shorter in the improvement group than in the non-improvement group, but the difference was not significant. As for patients’ distribution, there were no significant differences in suspected causative microorganisms between the two groups. Actual mortality rate in the improvement group (50%) was not significantly different from the predicted mortality rate by APACHE II score (48.9%).

**Table 1:** **Patient characteristics at baseline**

**Case number**	**78 patients**
Sex (male/female)	50/28
Age (years)	70 (20–87)
APACHE II score	24 (3–38)
SOFA score	11 (0–19)
MAP before the PMX treatment (mmHg)	64 (40–104)
CVP before the PMX treatment (mmHg)	15 (12–22)
Creatinine before the PMX treatment (mg/dl)	1.42 (0.41–10.24)
Endotoxin level before the PMX treatment (pg/mL)	11.3 (0.8–1925)
Lactate level before the PMX treatment (mmol/L)	3.7 (1.0–23.0)
Patients with diabetes mellitus	24
Patients received corticosteroid therapy	13
The times of PMX therapy	1 (1–6)
Total PMX treatment time (min)	1456 (80–18,100)
Suspected causative microorganisms	
GNR	41
GPC	12
GPR	2
Unknown origin	23
Infection site	
Abdomen	27
Bloodstream	15
Lung	10
Urinary tract	6
Others (unknown origin)	20

Data are presented as number of cases or median (range). *MAP* mean arterial pressure, *CVP* central venous pressure, *APACHE II* Acute Physiology and Chronic Health Evaluation II, *SOFA* Sequential Organ Failure Assessment, *PMX* polymyxin B-immobilized fiber column hemoperfusion, *GNR* Gram-negative rods, *GPC* Gram-positive cocci, *GPR* Gram-positive rods.

**Table 2:** **Changes in inotropic score and PaO**_**2**_ **/F**_**I**_ **O**_**2**_ **ratio from pre-PMX to post-PMX**

	**pre-PMX**	**post-PMX**	***p*** **value**
Inotropic score	18.1 (24.6)	9.9 (19.0)	0.011
PaO_2_/F_I_O_2_ ratio	201 (220)	205 (203)	0.956
Vasopressor dependency index (/mmHg)	0.27 (0.34)	0.14 (0.27)	0.002

Data are presented as median. *IQR* interquartile range, *PMX* polymyxin B-immobilized fiber column hemoperfusion.

**Table 3:** **Patient characteristics of improvement group and non-improvement group**

	**Improvement (** ***n*** **= 42)**	**Non-improvement (** ***n*** **= 36)**	***p*** **value**
Sex (male/female)	24/18	26/10	0.237
Age (years)	69 (13)	71 (34)	0.996
APACHE II score	24 (12)	24 (12)	0.980
SOFA score	11 (4)	11 (7)	0.996
MAP at baseline (mmHg)	62 (10)	72 (22)	0.343
Creatinine at baseline (mg/dl)	1.28 (1.72)	1.65 (1.85)	1.000
Lactate level at baseline (mmol/L)	3.2 (5.9)	4.2 (4.9)	0.599
Patients with diabetes mellitus	13	11	1.000
Patients received corticosteroid therapy	8	5	0.762
The times of PMX therapy	1 (1)	1 (1)	0.281
Total PMX treatment time (min)	1598 (2972)	1412 (2254)	0.237
Inotropic score at pre-PMX	24.7 (21.0)	10.3 (18.6)	0.001
Inotropic score at post-PMX	6.8 (15.6)	16.6 (32.8)	0.037
Vasopressor dependency index at pre-PMX (/mmHg)	0.33 (0.44)	0.19 (0.29)	0.019
Vasopressor dependency index at post-PMX(/mmHg)	0.13 (0.26)	0.14 (0.28)	0.845
PaO_2_/F_I_O_2_ ratio at pre-PMX	210 (234)	199 (206)	0.980
PaO_2_/F_I_O_2_ ratio at post-PMX	254 (181)	172 (156)	0.041
Endotoxin level before the PMX treatment (pg/mL)	15.8 (60.5)	6.1 (23.7)	0.195
Endotoxin level after the PMX treatment (pg/mL)	1.4 (18.2)	0.8 (0.7)	0.267
Suspected causative microorganisms (GNR/GPC/GPR/unknown origin)	24/6/2/10	17/6/0/13	0.428
Median survival time (days)	72	131	0.172
Ventilator-dependent time (days)	13 (33)	7 (42)	0.557

Data are presented as number of cases or median (IQR). *IQR* interquartile range, *MAP* mean arterial pressure, *APACHE II* Acute Physiology and Chronic Health Evaluation II, *SOFA* Sequential Organ Failure Assessment, *PMX* polymyxin B-immobilized fiber column hemoperfusion, *GNR* Gram-negative rod, *GPC* Gram-positive cocci, *GPR* Gram-positive rods.

## Discussion

The present study showed that the PMX treatment significantly improved the median inotropic score and the vasopressor dependency index. The inotropic score has been referred to as the vasopressor score [15], and these scores are used for the vasopressor requirement. Dopamine and norepinephrine have been used as vasopressor therapy to restore and maintain blood pressure, although the use of dopamine was associated with a greater number of adverse events [16]. Both these agents influence alpha-adrenergic and beta-adrenergic receptors, but to different degrees. Alpha-adrenergic effect of norepinephrine is greater than that of dopamine. In the present study, more than half of the patients were treated with norepinephrine/dopamine or norepinephrine/dopamine/dobutamine, but few patients were treated with norepinephrine alone, suggesting that inotropic score more reflects the degree of vasodilation rather than norepinephrine alone. Therefore, we adopted inotropic score rather than norepinephrine alone. We assessed hemodynamics by dividing all of patients into two groups, those whose inotropic scores improved after the PMX treatment (improvement group) and those whose inotropic scores did not improve (non-improvement group). Next, we compared several factors between the two groups to identify which factors contributed to the improvement. We found no significant difference between the groups when we compared the status of immunosuppressive conditions by assessing, for example, whether the patients received corticosteroid therapy or had any history of diabetes mellitus or organ dysfunction. There were no significant differences between the two groups when we compared suspected causative microorganisms and endotoxin levels. We also found, however, that the inotropic score before the PMX treatment was significantly higher in the improvement group than in the non-improvement group. This was unexpected, as we had hypothesized that a better condition before the PMX treatment brought about stronger effects on hemodynamic status. The somewhat comparable MAP values in the two groups indicated that the improvement group required more catecholamines to achieve a blood pressure equivalent to that of the non-improvement group. In fact, the vasopressor dependency index before the PMX treatment was significantly higher in the improvement group than in the non-improvement group. Higher vasopressor dependency index means that more vasopressors are required to increase 1 mmHg of MAP, indicating peripheral vasodilatation. Hence, the peripheral blood vessels are likely to have been more dilated in the improvement group. Septic shock has two different phases: warm (or hyperdynamic) shock and cold (or hypodynamic) shock. Warm shock is characterized by high cardiac output and low peripheral vascular resistance. Therefore, the improvement group may include many patients with warm shock. The inotropic score was also found to decrease significantly after the PMX treatment in the improvement group. Of course, there may be some other possible mechanisms for improvement of hypotension. First, there may be sepsis induced-cardiomyopathy. Although the patients who required dobutamine were not so many, we did not examine echocardiography in all patients. Accordingly, we cannot completely deny sepsis-induced cardiomyopathy. Second, there may be increased permeability-induced intravascular hypovolemia. In early stage of sepsis, most of the patients had received a lot of fluid for resuscitation. Then, catecholamines were administered if the patients had continuous hypotension. As shown in Table 1, central venous pressure (CVP) was not low before the PMX treatment. Therefore, it seems unlikely that intravascular hypovolemia exists in all patients before PMX treatment. These findings, taken together, confirm that the PMX treatment was one of the effective factors in constricting the peripheral blood vessels. Earlier reports attribute the hemodynamic improvement from PMX treatment to the adsorption of anandamide (ANA) [4,17], 2-arachidonylglycerol (2-AG) [17,18], and nitric oxide (NO) [19]. ANA and 2-AG, two different endogenous cannabinoids, can be produced by activated macrophages and platelets, respectively [17]. ANA and 2-AG may be paracrine mediators of hypotension during shock that act via cannabinoid receptor type 1 (CB1), a cannabinoid receptor subtype localized in the peripheral vasculature. Several studies have demonstrated that ANA and 2-AG can elicit CB1-receptor-mediated hypotension in rats [4]. PMX is known to adsorb not only endotoxin, but also ANA, a molecule that induces hypotension during endotoxin shock. Recent *in vitro* findings have demonstrated that ANA in saline/ethanol and in serum is efficiently adsorbed in a column of polymyxin B-immobilized fiber [18]. The mechanism of interaction between ANA and polymyxin B is believed to be hydrophobicity [18]. NO is characterized as a major mediator responsible for vasodilation and a loss of vascular responsiveness to catecholamines in endotoxin shock [20]. Overproduction of NO by inducible NO synthase plays an important role in the pathophysiology of endotoxin shock [21]. NO is also produced by substances such as toxic shock syndrome toxin-1 (TSST-1), an exotoxin of *S. aureus*, or by lipoteichoic acid, a component of the cell wall of Gram-positive bacteria [22]. NO produced in large quantities by such mechanisms dilates the peripheral blood vessels and induces shock. PMX treatment reportedly decreases NO breakdown products (NOx) in urine [19]. As such, the inhibition of NO production by PMX treatment could prevent vasodilation and increase blood pressure.

Turning to clinical data, the first randomized controlled trial (RCT), a pilot study performed in six European academic medical centers in 2005, concluded that PMX treatment is associated with improved hemodynamic status and cardiac function [11]. As with previous reports, our findings suggest that endocannabinoids and NO, a vasodilator substance, are involved in the development of septic shock, and that their removal with PMX confers the antihypotensive effect of PMX treatment.

The PaO_2_/F_I_O_2_ ratio did not significantly improve after PMX treatment in the study population overall. When we divided the patients into an improvement group and non-improvement group, the PaO_2_/F_I_O_2_ ratio before PMX treatment was similar between the two groups. After the PMX treatment, however, the PaO_2_/F_I_O_2_ ratio decreased in the non-improvement group and increased in the improvement group. Hence, we inferred a slight association between the PMX treatment and improved PaO_2_/F_I_O_2_ ratio. Previous studies reported that PMX treatment improved pulmonary oxygenation in addition to hemodynamics [10]. One study demonstrated significant negative correlations between the PaO_2_/F_I_O_2_ ratio and the concentrations of IL-8 and neutrophil elastase in patients with septic shock [23]. Another study identified PMX-induced decreases of metalloproteinase 1 concentrations in patients with acute respiratory distress syndrome [8]. The improved pulmonary oxygenation conferred by PMX treatment may therefore derive from decreases in adhesion molecules, IL-8, neutrophil elastase [23], metalloproteinase 9, or the tissue inhibitor of metalloproteinase 1 [8]. This remains speculative, however, as no mechanisms for such an action has been unraveled. The EUPHAS study, a multicenter RCT performed in ten Italian intensive care units in 2009, found that PMX treatment brought about significant improvements in MAP, vasopressor indication, organ failure severity, and the PaO_2_/F_I_O_2_ ratio (*p* = 0.049) [10].

PMX treatment had no significant effects on ventilator-dependent time or survival time in this study, though the hemodynamics clearly improved after the treatment in the comparative analysis between the improvement and non-improvement groups. We were fairly certain that systemic treatments to improve other organ failure, inhibit inflammation, etc. were required for the treatment of septic shock in our patients, while PMX was helpful for improving hemodynamics.

Readers should be aware of several limitations of this study. First, medical technologies and treatment modalities changed considerably over the approximately 9 years intervening between the first and last hospitalizations of the study subjects. Intensive care techniques, drugs, and various medical devices all advanced during this period [1], and an influential report advocating a longer duration of PMX was published [13]. These changes during the long period of patient collection were very likely to have affected the data for this study. Second, this study was retrospective. There are ethical and moral perils in allocating patients without PMX treatment, as PMX treatment for septic shock is covered under the national health insurance system in Japan. This disallows randomized control trials in Japan; hence, many studies from Japan are retrospective case series with no control groups. The efficacy of PMX treatment is being evaluated in two more multicenter prospective RCT now underway, ABDO-MIX in Europe and EUPHRATES in the USA [24]. The ABDO-MIX study (ClinicalTrials.gov NCT01222663) is a French RCT that evaluated 28-day mortality in patients with septic shock due to peritonitis. Eligible patients (*n* = 240) were randomized to standard care versus standard care plus PMX treatment within 36 h of abdominal surgery to repair intestinal perforation. Although there was no significant difference in mortality between the groups, the study had a number of potential problems such as cartridge clotting and higher failure rates. Therefore, these factors may decrease the power to detect a difference [25]. We are eager to learn the results of the EUPHRATES study.

## Conclusions

In sepsis cases, because of the peripheral blood vessels dilation, the higher inotropic score means more requirement of catecholamine. Our study suggested that PMX treatment mainly helps for improvement of the hemodynamics of septic shock patients. Particularly good effects seemed to be achieved when peripheral blood vessels were excessively dilated due to septic shock.

## Abbreviations

PMX: Polymyxin B-immobilized fiber column hemoperfusion
APACHE: Acute physiology and chronic health evaluation
SOFA: Sequential organ failure assessment
MAP: Mean arterial pressure
LPS: Lipopolysaccharide
IL-6: Interleukin-6
TNF-α: Tumor necrosis factor
ICU: Intensive care unit
CIs: Confidence intervals
GNR: Gram-negative rods
GPC: Gram-positive cocci
MRSA: Methicillin-resistant *Staphylococcus aureus*
GPR: Gram-positive rods
CVP: Central venous pressure
ANA: Anandamide
NO: Nitric oxide
CB1: Cannabinoid receptor type 1
TSST-1: Toxic shock syndrome toxin-1
NOx: NO breakdown products
RCT: Randomized controlled trial
IQR: Interquartile range
